# Supplementary figures and images for: Diversity and distribution of Symbiodiniaceae detected on coral reefs of Lombok, Indonesia using environmental DNA metabarcoding
Source: PeerJ. 2022 Oct 24;10:e14006. doi: 10.7717/peerj.14006 (PMC9610659; doi:10.7717/peerj.14006)

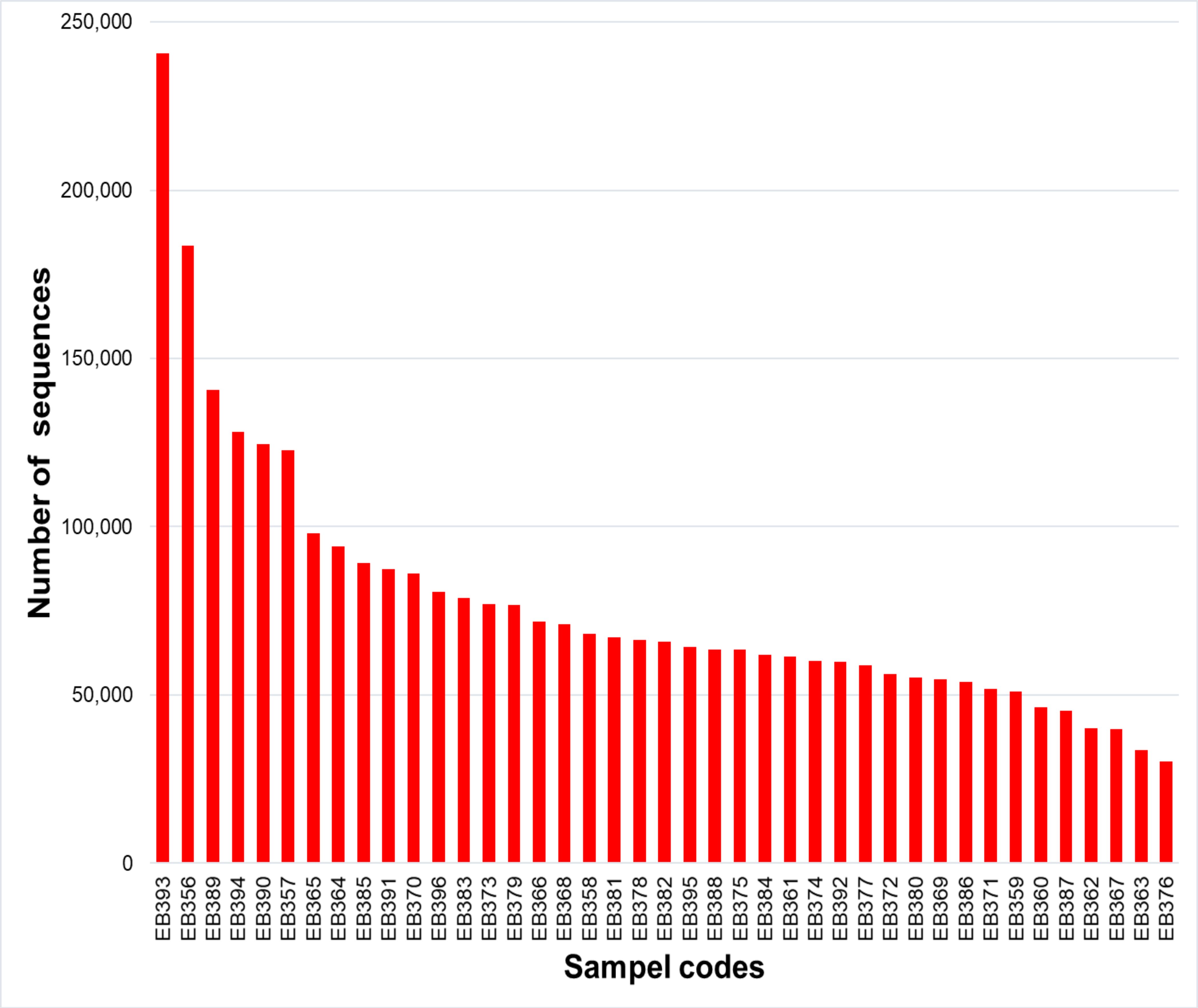

Supplement: Figure S1 [file peerj-10-14006-s001.png]
