# Supplementary material for: Diversity and distribution of Symbiodiniaceae detected on coral reefs of Lombok, Indonesia using environmental DNA metabarcoding
Source: PeerJ. 2022 Oct 24;10:e14006. doi: 10.7717/peerj.14006 (PMC9610659; doi:10.7717/peerj.14006)
Supplement: File S6 [file peerj-10-14006-s007.docx]

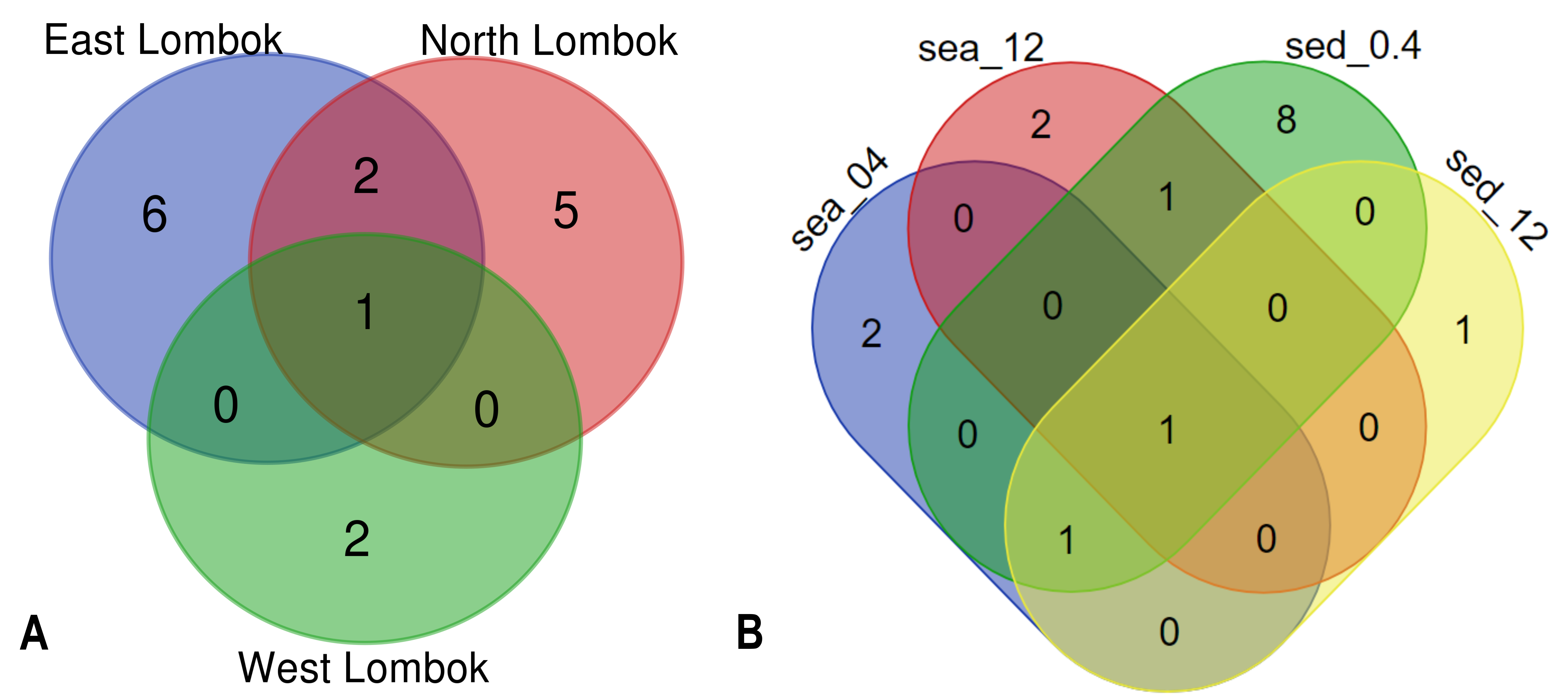


**Venn diagram of Symbiodiniaceae subclades around Lombok by: (A) coastal area and (B) method (sample type-filter pore size combination).** For a more detailed explanation of this figure, see table below. Sample labels: sea = seawater sample; sed = sediment sample; _0.4 and _12 indicate the pore size of the filter (in μm).

**Detailed explanation of unique elements at Venn diagram**.

| **Figures** | **Intersection** | **Total** | **Elements** |
| --- | --- | --- | --- |
| Fig. 4A | East_Lombok /North_Lombok/West_Lombok | 1 | C.sym1 |
|  | East_Lombok /North_Lombok | 2 | G.sym4 |
|  |  |  | D.sym2 |
|  | East_Lombok | 6 | D.sym6 |
|  |  |  | C.sym8 |
|  |  |  | C.sym7 |
|  |  |  | Csym16 |
|  |  |  | C.sym17 |
|  |  |  | C.sym10 |
|  | North_Lombok | 5 | C.sym12 |
|  |  |  | D.sym19 |
|  |  |  | D.sym22 |
|  |  |  | C.sym15 |
|  |  |  | B.sym20 |
|  | West_Lombok | 2 | S.sym21 |
|  |  |  | C.sym5 |
| Fig. 4B | sea_0.4/sea_12/sed_0.4/ sed_12 | 1 | D.sym2 |
|  | sea_0.4/sed_0.4/sed_12 | 1 | C.sym1 |
|  | sea_12/sed_0.4 | 1 | G.sym4 |
|  | sea_0.4 | 2 | D.sym19 |
|  |  |  | C.sym16 |
|  | sea_12 | 2 | H.sym12 |
|  |  |  | D.sym6 |
|  | sed_0.4 | 8 | C.sym7 |
|  |  |  | C.sym17 |
|  |  |  | C.sym5 |
|  |  |  | D.sym22 |
|  |  |  | C.sym15 |
|  |  |  | B.sym20 |
|  |  |  | S.sym21 |
|  |  |  | C.sym10 |
|  | sed_12 | 1 | C.sym8 |
