# Supplementary material for: Diversity and distribution of Symbiodiniaceae detected on coral reefs of Lombok, Indonesia using environmental DNA metabarcoding
Source: PeerJ. 2022 Oct 24;10:e14006. doi: 10.7717/peerj.14006 (PMC9610659; doi:10.7717/peerj.14006)
Supplement: File S7 [file peerj-10-14006-s008.docx]

**Prensence of Symbiodiniaceae based on site-medium-fraction sample combination.**

Sample label: ESea0.4 indicate site-sample type-filter pore size combination of East Lombok_Sea Water_0.4-12 μm; ESea12: East Lombok_Sea Water_>12 μm; ESed0.4: East Lombok_Sediment_0.4-12 μm; ESed12: East Lombok_Sediment_>12 μm; NSea0.4: North Lombok_Sea Water_0.4-12 μm; NSea12: North Lombok_ Sea Water_>12 μm; NSed0.4: North Lombok_Sediment_0.4-12 μm; NSed12: North Lombok_Sediment_>12 μm; WSed0.4: West Lombok_Sediment_0.4-12 μm.

|  | **A.Sym_21** | **B.Sym_18** | **C.Sym_1** | **C.Sym_5** | **C.Sym_7** | **C.Sym_8** | **C.Sym_10** | **C.Sym_15** | **C.Sym_16** | **C.Sym_17** | **D1.Sym_2** | **D1.Sym_6** | **D1.Sym_19** | **D1.Sym_22** | **G2.Sym_4** | **H.Sym_12** |
| --- | --- | --- | --- | --- | --- | --- | --- | --- | --- | --- | --- | --- | --- | --- | --- | --- |
| Esea12 | 0 | 0 | 0 | 0 | 0 | 0 | 0 | 0 | 0 | 0 | 1 | 1 | 0 | 0 | 1 | 0 |
| Esea0.4 | 0 | 0 | 1 | 0 | 0 | 0 | 0 | 0 | 1 | 0 | 0 | 0 | 0 | 0 | 0 | 0 |
| Esed12 | 0 | 0 | 1 | 0 | 0 | 1 | 0 | 0 | 0 | 0 | 1 | 0 | 0 | 0 | 0 | 0 |
| Esed0.4 | 0 | 0 | 1 | 0 | 1 | 0 | 1 | 0 | 0 | 1 | 0 | 0 | 0 | 0 | 1 | 0 |
| Nsea12 | 0 | 0 | 0 | 0 | 0 | 0 | 0 | 0 | 0 | 0 | 0 | 0 | 0 | 0 | 0 | 1 |
| Nsea0.4 | 0 | 0 | 1 | 0 | 0 | 0 | 0 | 0 | 0 | 0 | 1 | 0 | 1 | 0 | 0 | 0 |
| Nsed12 | 0 | 0 | 1 | 0 | 0 | 0 | 0 | 0 | 0 | 0 | 0 | 0 | 0 | 0 | 0 | 0 |
| Nsed0.4 | 0 | 1 | 1 | 0 | 0 | 0 | 0 | 1 | 0 | 0 | 1 | 0 | 0 | 1 | 1 | 0 |
| Wsed0.4 | 1 | 0 | 1 | 1 | 0 | 0 | 0 | 0 | 0 | 0 | 0 | 0 | 0 | 0 | 0 | 0 |
| **Sum** | 1 | 1 | 7 | 1 | 1 | 1 | 1 | 1 | 1 | 1 | 4 | 1 | 1 | 1 | 3 | 1 |
| **Perc. (%)** | **11.11** | **11.11** | **77.78** | **11.11** | **11.11** | **11.11** | **11.11** | **11.11** | **11.11** | **11.11** | **44.44** | **11.11** | **11.11** | **11.11** | **33.33** | **11.11** |
